# Supplementary material for: Are carbon emissions trading and green financial instruments synergistic? -Comprehensive quantitative research based on content analysis
Source: PLoS One. 2024 Mar 7;19(3):e0298601. doi: 10.1371/journal.pone.0298601 (PMC10919601; doi:10.1371/journal.pone.0298601)
Supplement: S1 Data — (ZIP) [file pone.0298601.s001.zip › Data/地级市Z_F工作报告.zip!/░▓╗╒/╗┤─╧╩╨/└┤╫╘╣1⁄2╓┌║┼í╛┬φ┐╦╩2╛▌═°í┐_2014.docx]

各位代表：
现在，我代表市人民政府，向大会报告工作，请予审议，并请市政协委员和其他列席人员提出意见。
一、坚持克难奋进，实现本届政府良好开局
刚刚过去的2013年，是宏观形势错综复杂的一年，也是淮南转型发展面临困难较多的一年。我们紧紧围绕科学发展主题，统筹稳增长、调结构、促改革、惠民生、保稳定，经济社会发展稳中有进。初步核算，全市地区生产总值820亿元，增长10%；财政收入170.1亿元，增长2.4%；固定资产投资804亿元，增长25.7%；社会消费品零售总额291亿元，增长13.8%；进出口总额5.1亿美元，增长44.7%；城镇居民人均可支配收入22900元，增长10.5%；农民人均纯收入8854元，增长13%；居民消费价格涨幅2.6%，基本完成市十五届人大一次会议确定的主要目标任务。
一年来，重点做了以下工作：
（一）全力稳定增长，经济运行平稳有序。面对严峻形势，我们把握大势，主动作为，有效施策。坚持扩大项目投资稳增长。实行经济工作项目化、项目工作责任化，强化重大项目调度，扩大有效投入。在建项目1270个，竣工776个，分别增长49.8%、45.1%。列入省“861”行动计划项目326个。煤制天然气项目前期有序开展，中安煤化、平电三期等项目加快推进，大气环境科技产业园一期、1000千伏特高压、淮南剧院等项目全面建成，淮浙电厂二期两台、淮沪电厂二期一台机组并网发电。坚持提升消费稳增长。把加快服务业发展与促进消费结合起来，服务业增加值占地区生产总值比重提高1.4个百分点。净增限额以上商贸企业114家。建成电商平台4家，实现电子商务交易额538亿元。金融、文化产业增加值分别增长9.3%、15%，旅游收入增长25.6%。开工商住房400万平方米，竣工150万平方米。农业服务业加快发展，实现农家店乡村全覆盖。坚持扶持实体稳增长。大力开展市领导帮扶重点企业、百家单位服务千户企业、百名干部挂职企业等活动。出台促进中小微工业企业健康发展26条等政策。安排支持经济结构调整和转型升级专项资金1.3亿元。落实“营改增”试点、企业社保减免等优惠政策，为企业减负2亿元。以上举措，有力地提振了市场信心，促进了经济平稳运行。
（二）致力转变方式，产业结构加快调整。适应转型发展的迫切要求，增强发展活力，夯实转型基础。工业结构加快优化。实施规模工业“双增”计划，净增规模以上非煤电工业企业249家，实现规模以上工业增加值465.5亿元，增长11.5%。非煤电工业、制造业占工业增加值的比重分别提高3.6个、3.5个百分点，战略性新兴产业产值增长14.2%。原煤产量8486万吨，下降7.1%；发电量537.8亿千瓦时，增长1.7%。民营经济加快培育。实施民营经济“双万”计划，净增民营企业3545家、个体工商户11609家。民营经济增加值占地区生产总值比重达43%，提高2个百分点。现代农业加快发展。全市“三农”支出32.5亿元，增长15.2%。全年粮食总产26.8亿斤，实现“十连增”。新增流转土地6.3万亩、设施农业1.2万亩。新增规模以上农产品加工企业53家、农民合作社184个，注册家庭农场85家。规模以上农产品加工企业实现产值151亿元，增长36.5%。农业社会化服务水平居全省前列。创新能力加快提升。科技支出占财政支出的比重达1.95%。新增高新技术企业10家、高新技术产品45个。授权专利1250件，其中发明专利133件。引进国外专家73人、硕士以上人才104人。国家能源局批准在淮成立煤炭开采国家工程技术研究院。全国能源行业煤矿瓦斯治理与利用标准化技术委员会在淮设立。煤化工产品质检中心通过国家认证。参与起草国家、行业、地方标准12项。实施股权激励试点企业2家。跻身全国智慧城市试点。第八次荣获全国科技进步先进市。
（三）合力抓好建设，城乡面貌展现新姿。把新型城镇化作为重要引擎，努力推动城乡协调发展。不断完善城乡规划。主城区控规实现全覆盖，东部新区产城一体化、中心村建设、产业发展等规划编制完成。依法整治建设环境。严厉打击违法建设行为，拆除违法建设447万平方米，推动征迁481万平方米，城市建设秩序明显好转。加强用地督察，规范土地市场。清理土地1878亩，新增耕地11052亩。持续提升城市功能。完成城乡建设投资295亿元。实施棚户区、老城区改造项目46个，启动集聚区规划建设。有序推进山南新区发展，青桐大道等7条道路竣工，泰丰大街等4条道路开工建设，市级公租房、安置房、职教园区等项目加快推进。孔李淮河大桥、淮上淮河大桥开工建设，舜耕山环山路、洞山东路等新改建项目基本建成，淮舜南路、夏郢西路、南山路、基地小街改扩建等工程竣工。新改建地下管网100公里。出台城市公共交通优先发展行动计划。投放新公交车300辆，新增、调整线路5条。加强工程车辆运输安全管理，全面取缔城市中巴车。加快建设美好乡村。23个省级重点示范村全面建成。“三线四边”环境综合整治在全省率先启动。完成农村危房和农房改造13929户。全年脱贫2.08万人。永幸河泵站重建、禹王站更新改造等主体工程完工。防汛抗旱工作成效明显，防洪排涝水平进一步提升。
（四）着力改革开放，发展活力不断增强。面对前进中的困难和约束，更加注重靠改革创新拓展空间。各项改革不断深化。平稳有序完成部分区域托管移交工作。园区等管理体制进一步理顺。严格依法招投标，建立政府性投资项目造价管理制度，四大类公共资源全部进场交易。大八公山旅游资源整合利用改革深入推进。创新组建市级融资租赁公司。成功发行首笔中小企业私募债。凤台农村金融综合改革试点取得突破性进展。开放合作不断拓展。纳入全国老工业基地调整改造规划。正式成为长三角城市经济协调会成员。积极推进合淮一体化进程。主动融入淮河生态经济带。与金融机构战略合作持续深化，全社会融资规模增加200亿元。海关、国检作用进一步发挥，通关更加便捷高效。招商引资不断加强。制定加快开发区转型发展的决定。建立优化投资环境五个机制。开展工业地产招商，开工建设工业地产160万平方米、竣工40万平方米。成功举办中国豆腐文化节。市经开区升格为国家级开发区，国瑞药业一期等项目建成投产。新型煤化工基地总体发展规划和产业发展规划编制完成，中安煤化项目完成投资30.3亿元。高新区完成投资15亿元，润敏江生物科技园等开工建设。全年引进内资700亿元，增长22.8%；利用外商直接投资2.4亿美元，增长26.4%。
（五）聚力社会治理，公共服务持续优化。以公共服务均等化为目标，不断优化资源配置。文化强市加快建设。《大地欢歌》、《千里长淮一条线》荣获中国第十届艺术节“群星奖”，少儿艺术获评国家公共文化服务体系示范项目，创作汇演大型花鼓灯歌舞《淮河风》。乡镇文化体育站实现全覆盖。广播影视、文学艺术进一步繁荣。文明创建持续加强。荣获全国见义勇为“城市奖”、省双拥模范城“八连冠”。省级文明城市创建取得积极成效。圆满完成市第三届道德模范评选。全市有6人入选“中国好人榜”，9人当选省第三届道德模范。社会治理深入推进。安全生产事故起数和死亡人数实现“双下降”。依法关停小煤矿8对、非煤矿山企业22家。食品药品监管力度持续加大。数字城管扎实推进，城管志愿服务经验被外地广泛借鉴。完善社会治安防控体系，平安淮南建设不断深化，社会保持和谐稳定。各项事业加快发展。成功创建全国中小学心理健康教育示范区、2个省级义务教育基本均衡发展县区。圆满完成学前教育三年行动计划，义务教育标准化学校建成率居全省前列。安徽理工大学新校区、淮师附小新校区加快建设。深化医药卫生体制改革。市一院门急诊综合楼、市四院病房大楼等开工建设。成功举办市第七届运动会。人口自然增长率6.47‰。第三次经济普查扎实推进。民族宗教、外事侨务工作有序开展，气象、防震、减灾、档案、地方志工作水平不断提升，妇女、儿童、残疾人事业取得进步，国防动员、人民防空、民兵预备役工作继续加强。
（六）努力保护环境，生态建设扎实推进。把生态作为最公平的产品，努力满足群众生态环境需求。节能减排目标任务全面完成。在全国率先出台资源环境审计监督办法。大力推进电力企业脱硫脱硝工程。实施节能与资源综合利用重点项目41个，潘集污水处理厂投入运行。化学需氧量、氨氮、二氧化硫和氮氧化物完成省下达的目标任务。采煤塌陷区综合治理扎实推进。投入资金12.1亿元，新开工安置点6个、竣工4个，搬迁安置居民1.55万人。治理塌陷区2平方公里。生态建设蓬勃开展。成功创建国家绿化模范城市。启动全国森林城市创建，超额完成森林增长工程年度任务。荣获省级生态村6个、生态乡镇4个、绿色生态城区1个。
（七）强力保障民生，人民生活有效改善。全市民生支出134.5亿元，占财政总支出的79.7%。民生工程取得新进展。投入资金20.2亿元，完成35项民生工程建设任务。开工建设保障性住房1.47万套，基本建成9700套。利用住房公积金贷款5.1亿元，支持保障性住房建设。就业创业实现新突破。开创性推进高校毕业生就业工作，实施大学生就学就业“万人计划”，成功引进大学生3000余名。建立城乡创业园18家。城镇新增就业4.8万人，城镇登记失业率控制在4%以内。转移农村劳动力2.2万人。社会保障得到新提升。各项社会保险参保319.3万人次。调整完善城镇医保、新农合政策，推进付费方式改革，建立大病保险制度。新农合参合率达103.6%。城乡低保、五保供养标准均提高10%以上。市社会福利保障区一期主体工程完工。
（八）大力改进作风，行政效能明显提升。不断优化政风行风，政务服务水平进一步提高。群众工作扎实推进。拓展网格化管理服务，及时解决群众反映强烈的热点难点问题。落实“四访一包”制度，畅通社情民意表达渠道。依法行政不断加强。办理市人大代表议案建议159件、政协委员提案472件。提请市人大审议地方性法规2件，制定政府规章2件。减少市级行政审批项目223项，精简率达56%，审批时限压缩54.3%。积极推行“一费制”改革。“12345”政府服务直通车开通运行。网络问政、政务公开不断加强，行政复议、法律援助有效开展。廉政建设力度加大。严格落实党风廉政建设责任制，进一步完善政府工作规则，大力整治“四风”、“三症”。坚持厉行节约、反对浪费，“三公”经费压缩20%。强化重点领域和关键环节的行政监察、审计监督，严厉查处违法违纪案件，塑造为民务实清廉的政府形象。
各位代表！这些成绩是在经济运行趋缓、发展要素趋紧的背景下取得的，是市委正确领导、科学决策的结果，是市人大依法监督、市政协民主监督的结果，是全市人民群策群力、共同奋斗的结果。在此，我代表市人民政府，向全市人民，向驻淮部队、武警官兵和政法干警，向各民主党派、各人民团体和社会各界人士，向关心支持淮南发展的同志们、朋友们、投资者和建设者，表示衷心的感谢！
在肯定成绩的同时，我们也清醒地看到，我市经济社会发展还存在不少矛盾和问题。主要表现为：一是转型步伐仍需加快。战略性新兴产业、现代服务业发展不足，园区经济、民营经济、县区经济发展需要提速。二是城乡功能仍需提升。区域之间、城乡之间资源配置不够均衡，公共服务水平还不够高，基础设施建设亟待加强。三是发展环境仍需优化。土地、资金、环保等要素制约加剧，生态建设、安全生产、信访稳定压力依然不小。四是政府职能仍需转变。“四风”问题仍不同程度存在，依法行政水平、政府工作效能需进一步提升。对此，我们将高度重视，认真加以解决。
二、全面深化改革，推动经济社会持续健康较快发展
今年是贯彻党的十八大精神、推进“五位一体”总布局的关键之年，也是落实十八届三中全会战略部署、全面深化改革的起步之年。当前，我们既要看到世界经济深度调整带来的压力，更应看到全球产业重新布局、国内经济质量效益向好、美好安徽建设势头强劲、淮南转型发展前景美好等有利条件，特别是全国资源型城市可持续发展规划、中原经济区、淮河生态经济带、合淮一体等平台效应不断彰显，我们必须适应新形势，抢抓新机遇，推动新发展。
今年工作的总体要求是：全面贯彻落实党的十八大、十八届二中、三中全会精神，坚持稳中求进工作总基调，以全面深化改革为动力，以“四争”目标为引领，以煤基与非煤产业协调发展为路径，以保障改善民生为落脚点，紧紧扭住“双增双万”、重点项目、创新驱动、“三区改造”、城乡提升，促进经济持续健康较快发展、社会和谐稳定，建设美丽新型能源城。
经济社会发展主要预期目标是：地区生产总值增长10%左右，财政收入增长6%以上，规模以上工业增加值增长12%，固定资产投资增长25%左右，社会消费品零售总额增长14%，进出口总额增长13%，城镇居民人均可支配收入增长10%，农民人均纯收入增长13%，城镇登记失业率控制在4%以内，人口自然增长率控制在8.5‰以内，节能减排、安全生产完成年度目标。
全面深化改革，实现持续健康较快发展，关键要把握六点。
一要把深化改革与思想解放结合起来。冲破思想观念的障碍、突破利益固化的藩篱，首要的是解放思想。我们要以老黄牛的毅力、千里马的气势，做到“胆子大、步子稳，不抢跑、不拖延”。 
二要把深化改革与稳中求进结合起来。只有稳中求进，才能为改革提供有利条件。我们要以“钉钉子”的韧劲，把改革贯穿于稳增长、调结构、促转型之中，贯穿于深化煤化、重化非煤、强化高新、优化人才之中，打基础、管长远、增后劲。
三要把深化改革与社会治理结合起来。当前，淮南发展进入换挡期。面对矛盾交织、利益多元的格局，必须以“不到长城非好汉”的决心，实现公共服务均等化、群众利益最大化。
四要把深化改革与文化繁荣结合起来。积极践行社会主义核心价值观，充分挖掘淮南文化元素，大力提升淮南城市形象，弘扬豆腐故里的历史底蕴、能源城市的奉献精神。
五要把深化改革与生态文明结合起来。彰显“三山、三水”的生态特色，着力推进绿色发展、循环发展、低碳发展，“让居民望得见山、看得见水、记得住乡愁”。
六要把深化改革与转变职能结合起来。坚持依法行政、简政放权、优化职能、规范运行，做到不越底线、不碰红线、不触高压线，永葆人民公仆的青松气质、红梅品格、修竹胸怀。为此，重点做好8个方面工作。
（一）千方百计稳定经济增长
充分发挥投资的支撑作用、消费的基础作用、引资的关键作用，巩固经济稳定向好势头。
扩大有效投入。高度关注国家政策走向，实施一批具有全局性、基础性、战略性的重大项目。改善投资管理和服务，实行项目开工、竣工闭环管理，推进项目投资提速、提质、提效。全年完成工业投资400亿元、城乡建设投资350亿元以上。加强对百个重大项目、省“861”行动计划项目调度，开工建设煤制天然气、光电产业园等项目，加快中国移动（安徽）数据中心、潘集选煤厂等项目建设，力促淮沪电厂二期、朱集西矿等竣工投产。
培育新兴消费。坚持城市扩容与培育消费同步推进，改善消费环境。加强流通体系建设，打造城市商圈和便民消费圈，完善农村消费服务体系。大力培育信息消费，加快物联网、互联网、云计算等技术应用，助推“网商电商”成长，拓展城市“一卡通”服务，打造“江淮云”产业基地。实施“宽带中国”淮南工程，固定宽带家庭普及率达72%。创新休闲、健康、文化消费，拓展家政、养老等生活服务消费。落实国家调整收入分配政策，努力增加居民收入，提高消费能力。实现社会消费品零售总额330亿元以上。
狠抓招商引资。注重发挥煤电资源绝对优势、综合环境比较优势，促进引资、引技和引智相结合，着力提高招商引资质量。建立健全招商引资专家谈判、合同文本审查、专项资金监管等制度，进一步优化投资环境。围绕工业地产，开展产业链、产业群招商。围绕国有企业混合所有制改造，开展以商招商。围绕煤化工产业，开展定向招商。围绕中国豆腐文化节等节庆，开展会展招商。围绕发展总部经济，深化与央企省企民企外企合作。围绕技术、服务输出，支持企业走出去。推动设立海关、国检正式机构，新增进出口实绩企业10家。全年引进内资、利用外商直接投资继续保持快速增长。
（二）坚持不懈推动转型升级
持续优化存量、扩大增量、提升总量，促进经济结构协调和生产力布局优化。
统筹推进煤基与非煤产业发展。坚持立足煤、延伸煤、不唯煤、超越煤的转型发展理念，煤基产业增加值增长11%，非煤电工业增加值占比进一步提高。优化煤基产业。推动煤炭产业循环发展，综合利用煤炭伴生资源。推动电力产业清洁发展，推进新庄孜低热值煤电厂直供电试点，加快超超临界机组建设。推动煤化工产业集聚发展，延伸煤化工产业链。推动煤机装备产业成套发展，加快凯盛重工等项目建设。壮大非煤产业。推动战略性新兴产业规模发展，战略性新兴产业产值、高新技术产业增加值分别增长20%、13%以上。推动现代服务业集群发展，出台支持服务业集聚区建设的实施意见，推进2个省级集聚区建设。统筹发展生产性、生活性服务业，促进发展提速、比重提高、水平提升。净增限额以上商贸企业100家。实现旅游收入87.5亿元。开工商住房400万平方米，竣工150万平方米。三产增加值增长8%以上。
统筹实施“双增”与“双万”计划。大力培育市场主体，激发发展活力。实施规模工业“双增”计划。落实工业化与信息化深度融合五年行动计划。实现规模以上工业增加值480亿元、非煤电工业企业主营业务收入540亿元，净增规模以上工业企业260家以上。实施民营经济“双万”计划。进一步落实省大力发展民营经济20条、市加快民营经济发展17条等政策措施，鼓励民间资本进入重点产业、基础产业、金融服务、社会服务等领域。净增民营企业4000家，新增个体工商户1.6万家，民营经济增加值占地区生产总值的比重达47.5%。
统筹提升县区与园区产业集中度。加强县区主要调度指标分类考核和经济运行监测。编制县区首位产业发展规划，各县区首位产业占比提高2个百分点。坚持产业集中、企业集群、土地集约、要素集聚，园区主导产业集中度达75%。开工工业地产400万平方米，竣工200万平方米。市经开区完成投资50亿元，引进项目30个以上，开工项目17个，竣工项目14个。加快煤化工园区建设，力争中安煤化项目完成投资100亿元。高新区完成投资20亿元以上，开工建设淮南谷科技研发孵化平台，培育生物医药、光电、信息等产业。支持县区工业园特色发展，探索产业配套互补的共赢模式。
（三）坚定不移深化改革创新 
处理好政府和市场的关系，使市场在资源配置中起决定性作用和更好发挥政府作用，最大程度释放改革红利。
深化重点领域改革。积极发展混合所有制经济，支持企业交叉持股、融合发展。推进20家企业股份制改造、2家企业改制重组，力争2家企业上市、2家企业在“新三板”挂牌。把政府性债务纳入全口径预算管理，有效防控债务风险。深化凤台县农村综合改革，开展农村产权制度改革试点。完善并推广粮食银行，探索建立土地银行。推进区划调整，确保各项工作有序平稳衔接。加快户籍制度改革，有序推进农业转移人口市民化。认真落实省里部署的各项体制改革任务。
实施创新驱动战略。建立健全鼓励创新的体制机制，充分发挥企业创新主体作用，研究与试验发展经费占地区生产总值比重达1.5%。“两化”融合发展水平指数提高4个百分点。加大专利工作力度，提高发明专利申请量和拥有量。申报创新型试点企业3家、高新技术企业10家、高新技术产品30个。推进“云海战略”智慧城市规划实施，加快中科院淮南新能源研究中心、中科院大气所淮南研究院等建设。争创全国质量强市示范市、全国防爆电气知名产品示范区，启动省光伏产品质检中心建设，建成省煤矿安全仪器仪表质检中心。加强国家和行业标准制定。申请认定中国驰名商标1件。
破解发展要素制约。积极发展普惠金融，丰富金融市场层次。加大股权融资和债券发行力度，提高直接融资比重。全社会融资规模增加230亿元。积极开展融资租赁，规范发展担保公司和小额贷款公司。落实中原经济区“人地挂钩”政策。建立城乡统一的建设用地市场。完善土地节约集约利用的政策措施，规范土地储备、出让管理，严处各类土地违法违纪行为。争取增减挂钩周转指标2000亩，整治土地5000亩以上。
（四）积极稳妥推进新型城镇化
坚持以人为本，构筑以工促农、以城带乡、工农互惠、城乡一体的新型工农城乡关系。
注重规划引领。积极参与合肥经济圈城镇体系、皖北城镇群城镇体系规划编制。细化经济社会发展规划、城市建设总体规划、土地利用总体规划和矿区发展规划。启动合淮共建区、高铁南站片区、高新区扩区、商贸物流功能区、地下管网等规划。修编城市防洪排涝、集中供热、给排水、供气等专项规划。完善道路线网、集聚区规划。加强规划批后管理，维护规划的刚性和权威。
强化建设推动。按照“老城新城一样美”的要求，塑造“双百城市”的新形象。推进东进南聚、西优北联。开工建设滨湖大道、站前路，推进滨河大道建设。改造铁路下穿立交桥4座，推进2座排涝泵站建设。提升山南新区公共服务设施和基础设施配套水平，协调服务商杭高铁淮南段暨高铁南站建设，开工建设九龙大道、大别山引水工程，推进奥体公园等项目。大力优化西部城区路网水网管网和公共服务设施，实施洞山西路改造、沿矿路、谢一矿路建设。支持凤台、潘集、毛集联动发展，开工建设济祁高速公路淮南段、淮河二桥，推进孔李淮河大桥、淮上淮河大桥、凤台二桥等工程，启动凤毛快速通道前期工作。实施“三区改造”。开工建设7个集聚区、推进3个、做好前期工作2个。改造棚户区4.3万户、550万平方米。新开工采煤塌陷区居民安置点5个、竣工4个。
推进功能完善。进一步加大拆违力度，基本完成集中整治违法建设任务。开工建设城市畅通一环工程、城市轨道交通一号线试验段。实施主城区20米以下道路三年行动计划，新改建城区道路13万平方米。推进城市水系改造亚行贷款项目，建设2座污水泵站。改扩建各类管网130公里。调整改造省道6条。加强汽车客运站、公交首末站建设，投放新公交车200台，新辟公交线路6条。
（五）毫不动摇夯实“三农”基础
坚持以深化改革为动力，加大强农惠农富农政策力度，促进农业特色发展、农民安居乐业、农村经济繁荣。
构建新型农业经营体系。积极发展股份合作、土地合作，新增农民专业合作社150家、家庭农场100家、市级以上农业产业化龙头企业20家。规模以上农产品加工产值增长10%。加快现代农业示范园建设，新增流转土地5万亩。
提升农业综合生产能力。坚持稳定面积、主攻单产，力促粮食丰产丰收，切实提高粮食安全保障能力。加快农业科技进步，提升农业社会化服务水平。加强农产品质量检测监管，不断提高安全水平。加强农田水利基本建设，开工建设西淝河退堤工程和洼地治理项目，加快朱集大型泵站改造、中小河流治理，启动小型农田水利提升改造工程，完成重点洼地世行贷款项目。规范淮河河道采砂管理。
促进农民持续增收。强化对农民增收的政策支持，提高集约经营、规模经营、社会化服务水平。实施“农民增收行动”，力争农民人均纯收入突破1万元。加大扶贫脱贫工作力度，让广大农民过上更好的日子。
努力建设美好乡村。建立社会资本投向农村的引导机制，鼓励工商资本参与农村现代化建设。建成48个美好乡村中心村。完成3000户农房建设、1650户危房改造，建成11个乡镇农村清洁工程。推进“三线四边”环境综合整治，改善居民生活条件。
（六）齐心协力改善民生
坚持底线思维、突出重点、完善制度，让发展成果更多更公平地惠及全市人民。
巩固完善民生工程。加强民生工程绩效考核，健全建后管护长效机制。实施33项民生工程。开工建设保障性住房1.48万套，基本建成8000套。建成50个农民体育健身工程。解决农村15万人安全饮水问题。新建改建菜市场6个。完善“五位一体”便民服务体系。
推进教育优先发展。落实教育综合改革任务，大力推进素质教育。统筹教育资源配置，促进城乡、区域和各级各类教育均衡发展。创建省级义务教育基本均衡发展县区3个。建设标准化学校23所。新改扩建公办幼儿园30所。建成安徽理工大学新校区、职教园区、市体育学校、淮师附小新校区。加大引才引智力度，对口培养高技能人才。
争创国家创业型城市。深入实施大学生就学就业“万人计划”。加快创业园建设。完成就业技能培训1.9万人。城镇新增就业4万人，转移农村劳动力2万人，实现困难人员就业3000人。推行建筑施工企业农民工工资实名制，基本实现农民工工资无拖欠。
提高群众健康水平。积极争创省级卫生城市。继续推进县级公立医院综合改革。建成市一院门急诊综合楼、市四院病房大楼，开工建设市妇幼保健院新院等项目。鼓励发展专科医院和民办医院。健全农村医疗卫生服务体系，加强基层医技人员队伍建设。继续做好人口计生工作，提高优生优育水平。
完善社会保障体系。加强各项社会保险扩面征缴，整合城乡居民基本养老保险、医疗保险制度。开展行业社会保险移交地方工作。提高城镇职工、居民社会保险待遇标准。城乡低保、五保供养标准均增长10%以上，农村五保集中供养能力达55%以上。完善生活必需品储备调控办法，保持价格总水平基本稳定。
高度重视老龄工作。落实民办养老机构各项优惠政策，完善以居家养老为基础、社区养老为依托、机构养老为支撑的养老服务体系。推进农村敬老院升级改造，加快向养老服务中心转变。开工建设市社会福利保障区二期、社区日间照料中心60个，城市社区养老服务设施覆盖率达43%。
（七）持之以恒推进文化社会建设
积极推动文化繁荣发展和社会治理创新，最大限度增加和谐因素，确保社会安定有序。
提高文化软实力。加快社会主义核心价值体系建设，巩固省级文明城市创建成果。推进历史文化名城创建，启动历史文化街区开发保护工作。深化文化体制改革，完善文化管理体制和文化生产经营机制，建立健全现代公共文化服务体系、现代文化市场体系。不断繁荣文化事业，广泛开展“大地欢歌”等群众性文化活动，推进市文化艺术中心、市博物馆三期建设，加快县区图书馆、文化馆建设，建成公共电子阅览室6个。大力培育文化产业，推进印刷产业园、豆腐文化产业园建设，支持省级文化产业示范基地发展。大力实施“十个一工程”，推进文化精品创作、营销和开发。组团参加省第十三届运动会。
促进社会治理创新。全面加强系统治理、依法治理、综合治理、源头治理，完善人民调解、行政调解、司法调解联动体系。创新有效预防和化解社会矛盾体制，推进网格化、市场化、信息化、社会化管理服务。加强社会治安大巡防和防控体系建设，严厉打击各类违法犯罪活动。建立健全网上受理信访制度，及时解决群众合理诉求。推进社区管理体制改革，完善基层社会管理服务。启动第九届村民委员会换届选举。注重发挥工会、共青团、妇联等人民团体作用，引导广大群众参与平安淮南建设。 
健全公共安全体系。加强重点行业和领域安全隐患整治，依法关停小煤矿，严防各类事故发生。加强食品药品安全监管，营造公平有序的市场环境。加强道路交通整治，依法“打非治违”。加强应急平台和队伍建设。健全网络突发事件处置机制。加强国防动员和民兵预备役建设，推动军民融合深度发展。做好人民防空、民族宗教、防震减灾、残疾人、地方志、统计、气象和档案等工作，不断提升群众的满意度和幸福感。
（八）一以贯之优化生态环境
正确处理发展经济和保护环境的关系，构建资源节约型、环境友好型的生态体系，促进生产空间集约高效、生活空间宜居适度、生态空间山清水秀。
加强节能减排。开展国家环保模范城市创建，实施6个污水防治重点项目，抓好50个节能与资源综合利用重点项目、11座垃圾转运站等建设，完成6台火电机组脱硝改造。开展城区燃煤锅炉污染治理，综合整治非煤矿山和固体废弃物，加强农村污染防治和秸秆禁烧，有效减少雾霾危害。
加强生态修复。强化城市绿线管理，划定生态保护红线。加快八公山、舜耕山、上窑山山体修复，实施舜耕山环山路植被修复工程。启动江淮分水岭地区水系治理，提高瓦埠湖和高塘湖水质。加快焦岗湖生态湿地修复。推进凤凰湖水系治理。加大采煤塌陷区等生态脆弱地区环境修复力度。
加强生态文明建设。推进国家森林城市创建，完成森林增长工程10万亩。推进国家生态园林城市创建，实施城区绿荫提升工程，加强滨河公园、广场游园和城市绿道建设。打造舜耕山风景区，开工建设淮南子植物园和7大景点。推进八公山、卧龙山、茅仙洞绿色生态长廊建设。建设滨高塘湖森林景观带。推进淮河生态经济带建设，实施滨河森林景观带工程。坚持尊重自然、顺应自然、保护自然，努力让淮南人民在蓝天下、青山旁、绿水边、自然中幸福地生活。
三、切实转变职能，努力建设法治政府和服务型政府
经济社会发展的新形势、全面深化改革的新任务，要求我们发扬“雷厉风行、言行一致，严谨创新、敢于担当”的工作作风，谋好政府该谋的事、管好政府该管的事、干好政府该干的事。
一是坚持科学理政。按照“一张蓝图干到底”的要求，落实中央、省里和市委的决策部署，踏石留印、抓铁有痕、善做善成。自觉接受人大法律监督、政协民主监督、新闻舆论监督和群众社会监督，认真办理人大议案、代表建议和政协提案。坚持科学、依法、民主决策，完善重大决策、规范性文件和行政行为合法性审查程序。全面正确履行政府职能，强化公共服务、市场监管、社会治理、环境保护等职责。树立正确的考核导向，完善发展成果考核评价体系。
二是坚持依法行政。切实维护宪法法律权威，加强重点领域政府立法。深化行政执法体制改革，提高执法和服务水平。健全行政复议案件审理机制，纠正违法或不当行政行为。坚持用制度管权管事管人，探索建立政府及其工作部门权力清单制度。强化招投标监管体系建设，努力做到好中选优。进一步精简行政审批项目，积极推进网上审批。办好“12345”政府服务直通车。深入治理政风行风文风会风，从严查处“三乱”和吃拿卡要等行为。
三是坚持为民勤政。始终把群众利益放在第一位，注重解决事关群众切身利益的问题。扎实开展群众路线教育实践活动，深入一线查民情，深入基层解民忧，少说多做，实干实效。完善直接联系和服务群众制度，畅通群众诉求表达渠道。尊重群众首创精神，强化基层基础，激发基层活力。拓宽政府购买服务领域，最大范围引入竞争机制。大力整治“软、懒、散”现象，不断提升行政效能。
四是坚持廉洁从政。严格执行中央、省、市各项规定，加强廉政风险防控管理。扎实推进决策、管理、服务、结果公开，全面公开“三公”经费预决算，压缩“三公”经费10%。加强行政效能监察和审计监督，强化行政问责和绩效评估。坚决“把权力关进制度的笼子里”，形成不敢腐的惩戒机制、不能腐的防范机制、不易腐的保障机制，寻求改革共识的最大公约数，汇聚转型发展的强大正能量。
各位代表！十八大吹响了全面建成小康社会的新号角，十八届三中全会开启了全面深化改革的新征程。让我们紧密团结在以习近平同志为核心的党中央周围，在市委的坚强领导下，解放思想、改革开放，真抓实干、开拓创新，为建设生态环境优良、产业特色鲜明、人民生活幸福的美丽新型能源城而努力奋斗！
